# Factors Associated With Food Texture Acceptance in 4- to 36-Month-Old French Children: Findings From a Survey Study

**DOI:** 10.3389/fnut.2020.616484

**Published:** 2021-02-01

**Authors:** Carole Tournier, Lauriane Demonteil, Eléa Ksiazek, Agnès Marduel, Hugo Weenen, Sophie Nicklaus

**Affiliations:** ^1^Centre des Sciences du Goût et de l'Alimentation, AgroSup Dijon, CNRS, INRAE, Université Bourgogne Franche-Comté, Dijon, France; ^2^R&I, Blédina, Limonest, France; ^3^Danone Nutricia Research, Utrecht, Netherlands

**Keywords:** eating ability, chewing skills, infant, parental report-based measures, food texture, complementary feeding, feeding practices

## Abstract

Food texture plays an important role in food acceptance by young children, especially during the complementary feeding period. The factors driving infant acceptance of a variety of food textures are not well-known. This study summarizes maternal reports of children's ability to eat foods of different textures (here: acceptance) and associated factors. Mothers of 4- to 36-month-old children (*n* = 2,999) answered an online survey listing 188 food-texture combinations representing three texture levels: purees (T1), soft small pieces (T2), hard/large pieces, and double textures (T3). For each offered combination, they reported whether it was spat out or eaten with or without difficulty by the child. A global food texture acceptance score (TextAcc) was calculated for each child as an indicator of their ability to eat the offered textured foods. The results were computed by age class from 4–5 to 30–36 months. The ability to eat foods without difficulty increased with age and was ranked as follows: T1> T2 > T3 at all ages. TextAcc was positively associated with exposure to T2 (in the age classes between 6 and 18 months old) and T3 (6–29 months) and negatively associated with exposure to T1 (9–36 months). Children's developmental characteristics, as well as maternal feeding practices and feelings with regard to the introduction of solids, were associated with texture acceptance either directly or indirectly by modulating exposure. Children's ability to eat with their fingers, gagging frequency, and to a lesser extent, dentition as well as maternal feelings with regard to the introduction of solids were the major factors associated with acceptance. This survey provides a detailed description of the development of food texture acceptance over the complementary feeding period, confirms the importance of exposure to a variety of textures and identifies a number of additional person-related associated factors.

## Introduction

Early childhood is a period of rapid growth and plays a critical role in the development of health outcomes (1). Dietary experiences during this period are critical from both nutritional and developmental points of view because they shape eating habits during later childhood and even adulthood (2, 3). It is therefore important to fully understand the dietary experiences that promote healthy eating habits, especially during the complementary feeding (CF) period when a large variety of foods other than milk are introduced to the infant's diet. In this context, the development of food texture acceptance during the course of CF merits scrutiny. Indeed, the texture of food plays a crucial role in food rejection and contributes to feeding difficulties in children (4–7). Food texture acceptance develops with age throughout the course of the CF period and is related to the development of children's oral-motor skills. The development of these skills ensures an effective transition of the child's diet toward the foods from the family table. However, a detailed description of the development of children's acceptance across the CF period and for a variety of textures has not yet been provided.

Acceptance of food texture requires children to have the ability to chew and swallow food. Children's chewing has been investigated in experimental studies using different methods, such as the evaluation of video recordings of infants eating foods (4, 8, 9), the monitoring of chewing muscle activity and jaw movements (10–12) or the determination of particle sizes of boluses collected under standardized conditions (13). Children's acceptance has been determined from observation of ingestive behavior; a food is considered accepted when it is eaten by the child. The acceptance level of various textured foods was defined from the mean percentage of children of a given age swallowing a small quantity/piece of these foods (14) or from food intake (in grams or number of spoons) (7, 15, 16).

Various individual factors were suggested to influence food texture acceptance in infants and toddlers. The primary factor is the degree to which children have been exposed to a diet of varied food textures. Early exposure to a large variety of textures after the initiation of CF stimulates the development of oral-motor skills and facilitates the acceptance of more complex textures (15, 17–19). Other maternal feeding practices, such as breastfeeding or eating the same foods as the family, are also thought to be favorable for texture acceptance (20). Texture acceptance is also modulated by individual eating temperament (15, 16) and tactile sensitivity (21–23). Dentition is thought to play a role in the development of chewing ability in 9-to-36-month-old children (11), and the number of teeth was positively associated with the *ad-libitum* intake of chopped carrots among 12-month-old children (15). Finally, individual differences in developmental factors possibly associated with readiness to eat food pieces (e.g., ability to sit alone, ability to eat alone with fingers or with a fork) have not been studied specifically but probably also play a role in children's acceptance of solid foods.

Whereas, experimental studies are the most objective and controlled way to evaluate the development of food texture acceptance, they are limited to a small subset of foods and children (4, 14–16). In addition, the laboratory environment and/or the process of being observed may alter the child's eating behavior compared to the daily situation at home (24). The alternative is to study food texture acceptance in a survey, which does not have these disadvantages and allows a larger number of subjects. This makes it possible to study a number of factors together and compare them. A study based on parental reports of children's ability to eat specific foods would make the evaluation of texture acceptance possible (1) for the textured foods introduced to children's diet, (2) to compare eating ability at specific time points during the entire CF period, and (3) to assess factors associated with interindividual variability at a given age. Parental self-reports have been used in many studies to assess different facets of children's eating behavior [baby and children eating behavior (25, 26) or eating difficulties (6, 27)] with success. However, few attempts have been made to evaluate the ability to eat food texture using this approach. A previous study (28) conducted in-home interviews to assess the oral-motor development of children between 2 and 24 months. Mothers reported the child's age when specific behaviors (eating food with tiny lumps, chewing and swallowing firmer foods without choking, etc.) first occurred. Another previous study (29) used an open-ended survey for parental reports of food textures that are “easy” or “difficult” to eat for their child with Down syndrome. Finally, Sakashita et al. (20, 30) proposed a detailed questionnaire containing food items offered during CF in Japan for which parents evaluated their child's eating ability. To date, no questionnaire has been reported to assess children's ability to eat textured foods offered during CF in France, where children have been reported to be exposed to textured foods only to a limited extent before 12 months (31) and where texture introduction is a matter of concern for some parents (32).

The objective of this work is to evaluate the development of infants' and toddlers' ability to eat a variety of food textures using a cross-sectional study. In a previous publication (31), we reported data showing the course of introduction of foods in children aged between 4 and 36 months old. In the present work, we studied parental evaluation of their child's ability to eat the foods they introduced, representing a large range of textures. Specifically, the first objective is to report the evolution of food texture acceptance with age. The second objective is to study the individual factors associated with acceptance among children of a given age. It was hypothesized that older children display a better ability to eat foods with different textures than younger ones, that food acceptance (as reported by parents) would be positively associated with dietary exposure to a variety of textures and that acceptance would be positively related to children's number of deciduous teeth and feeding skills.

## Materials and Methods

Data were collected using a survey conducted with parents of French children aged 4–36 months, aiming to describe both parental feeding practices with regard to food texture introduction and texture acceptance by children. The cross-sectional survey was launched online through a large database of members of the web information programme of the Blédina brand [declared to the national data protection authority, the Commission Nationale Informatique et Liberté (CNIL), no. 1824320v0] from September to December 2015. The survey was approved by the local ethics committee (Comité de Protection des Personnes Est III, no. 2015-A00323-46).

### Description of the Survey

The first part of the survey collected information on maternal characteristics (age, country of birth, education level, source of information for advice on CF practices) and children's characteristics (sex, birth order, measured birth and current weight and length, number of teeth). Birth and current weight-for-length z-scores were determined using the World Health Organization child growth standards (33). Parents evaluated their children's motor skills (sitting up alone, pacifier use, thumb sucking, drooling) and feeding skills/behaviors (eating with fingers, self-feeding with a fork, gagging when food or object enter the mouth) using a 4-category scale: “never,” “rarely,” “sometimes,” and “often.” Reported maternal feeding practices included breastfeeding (yes/no), age at CF introduction, letting the child participate in family meals (yes/no), attendance to day care meals (yes/no), practice of baby-led weaning (BLW, yes/no) and the type of food preparation for their child (“exclusive use of ready-prepared baby food,” “exclusive use of homemade foods” or “use of both ready prepared and homemade foods”). Finally, maternal feelings (“eager,” “unconcerned,” “reluctant”) regarding the introduction of solid foods were reported.

The second part of the survey aimed to evaluate acceptance of the solid foods that children had already tried. This part was inspired by the survey developed by Sakashita and collaborator for Japan (20) and adapted to CF practices in France. Parents were shown a list of 61 foods commonly used in France, with each food item presented in different texture formats (puree, pieces, raw, cooked, etc.). A maximum of 188 combinations were shown (Supplementary Material 1). For example, for “carrot,” the following food-texture combinations were shown: smooth carrot puree, rough carrot puree, cooked carrot in small pieces, cooked carrot in large pieces, raw grated carrot, raw carrot in small pieces, and raw carrot in large pieces. To help parents in their assessments, they were provided with pictures illustrating the size of the pieces with a scale (Supplementary Material 2). For each food, mothers were asked to record whether they had already offered it to their child (yes/no). If they introduced the food, they self-reported for each food-texture combination introduced their child's ability to eat this combination by selecting one of the following answers: “*offered but spat out immediately,” “chewed but spat out,” “sucked and swallowed,” “eaten with some difficulties,” “eaten without difficulties*.” Preliminary analysis showed that some answer categories were rarely selected. Therefore, in the reporting and analysis, some categories were grouped together: “*offered but spat out immediately”* and “*chewed but spat out”* were grouped into “*spat out”* and coded as 0; “*sucked and swallowed”* and “*eaten with some difficulties”* were grouped into “*eaten with difficulties”* and coded as 1; “*eaten without difficulties”* was coded as 2.

### Definition of Food Texture Levels and Coding of Acceptance Answers

The 188 food-texture combinations were categorized into three texture levels according to the feeding skills necessary to process the food (see full list and level classification in Supplementary Material 1). Smooth and rough purees, which can be processed by sucking motions or limited tongue-palate compressions, were categorized as “simple texture,” also called the T1 level. Soft solid textures (small cooked pieces, soft foods) that require more intensive tongue-palate or gum-gum compressions were categorized as “intermediate texture” (T2 level). Last, large cooked and/or hard pieces that require the tongue, the presence of teeth and masticatory movements to be swallowed and double textures (pieces in a thin liquid phase), which require swallowing the liquid phase while maintaining the pieces in the oral cavity for further breakdown, were categorized as “hard/large pieces and double textures” (T3 level). By doing so, among the 188 food-texture combinations in the survey, 39 were classified at the T1 level, 40 at the T2 level and 109 at the T3 level (Supplementary Material 1).

### Determination of a Food Texture Acceptance Score (TextAcc)

For each child, we determined a food texture acceptance score (TextAcc, **Equation 1**), which is a global indicator of a child's ability to eat food textures and was aimed at comparing children of the same age and identifying factors of the observed differences. We designed the score in such a way that it increased with the level of acceptance (spat out < eaten with difficulties < eaten without difficulty) of given food and with the texture level of this food (T1 < T2 < T3). The score takes into consideration the total number of foods introduced in the child's diet [which is known to vary considerably among children of a given age class (31)]. This score was built as follows: first, the number of food-texture combinations offered to the child was determined for each texture level (NT1, NT2, NT3). Then, an acceptance score was calculated for each texture level from the sum of the acceptance levels (coded 0 {“spat out”}, 1 {“eaten with difficulties”} or 2 {“eaten without difficulties”}) of offered food-texture combinations. These scores were assigned a different weight, depending on the texture level: 1 for the T1 level, 2 for the T2 level, and 3 for the T3 level. TextAcc was finally obtained from the sum of the weighted acceptance scores collected for the T1, T2, and T3 levels divided by the total number of food-texture combinations offered to the child (**Equation 1**).

(1) $$TextAcc=\frac{\;\sum_{i=1}^{NT1}(acceptance\;level_{i}\times 1)+\sum_{j=1}^{NT2}\left(acceptance\;level_{j}\times 2\right)+\sum_{k=1}^{NT3}\left(acceptance\;level_{k}\times 3\right)}{NT1+NT2+NT3}$$

where T1 is the texture of smooth and rough purees, T2 is soft solid textures, and T3 is the texture of large cooked and/or hard pieces and double textures; i, j, and k: one food-texture combination within the texture levels T1, T2, and T3; NT1, NT2, NT3: the number of food-texture combinations of texture level T1, T2, and T3 offered to the child; acceptance level: acceptance level of a given food-texture combination (0: “spat out,” 1: “eaten with difficulties,” 2: “eaten without difficulties”).

### Statistical Analysis

Data were split into 14 age classes in agreement with (31). The split was organized by month during the first 12 months (except for infants of 4 and 5 months, which were grouped together), as the infant's oral skills develop quickly during this period. Above the age of 12 months, responses were split into larger age classes: 13–15, 16–18, 19–21, 22–24, 25–29, and 30–36 months.

Statistical analyses were run using SAS 9.4 (SAS Institute, Inc., Cary, North Carolina). For each age class, we determined the ratio (%) of food-texture acceptance “*spat out,” “eaten with difficulties,” “eaten without difficulties”* over the total number of combinations offered within each texture level (T1, T2, T3). The evolution of these ratios with age was assessed using one-way analyses of variance (ANOVAs) and Student Newman-Keuls *post-hoc* analyses to compare mean values. The impact of texture on the ratio was studied for each age class using one-way ANOVAs and Student Newman-Keuls *post-hoc* analyses.

The effect of age class on TextAcc was assessed using ANOVA and the Student-Newman-Keuls test *post-hoc* analysis. The study of factors associated with this score was performed for each age class independently. Associations between TextAcc and 20 variables representing children's characteristics (“sex,” “number of teeth,” “birth order,” “current weight-for-length z-score”), motor and feeding skills (“use of pacifier,” “thumb sucking,” “drooling,” “gagging,” “sitting alone,” “eating with fingers,” “self-feeding with a fork”) and maternal feeding practices (“breastfeeding,” “age of CF,” “T1 exposure score (number of T1 combinations introduced),” “T2 exposure score,” “T3 exposure score,” “attendance at day care meal,” “type of food preparation,” “meal taken with the family”) and “maternal feeling with regard to the introduction of solids” were studied using separate bivariate linear models. The results from bivariate analysis are presented in Supplementary Material 3. Variables significantly associated with TextAcc for at least four age classes were entered in a multivariate linear model, which included the number of T1, T2, and T3 foods introduced, number of teeth, eating with fingers, gagging, age of CF, and maternal feelings concerning the introduction of solid foods corrected for weight-for-length z-score.

## Results

### Study Population

A total of 3,771 respondents participated in the survey. Data from respondents other than mothers (fathers or grandmothers, *n* = 71), twins (*n* = 37), children born at a gestational age under 37 weeks of amenorrhea (*n* = 137), with severe gastroesophageal reflux (*n* = 247) or tube-fed at birth (*n* = 139), aged below 4 months or above 36 months (*n* = 131) and missing data with regard to food texture introduction (*n* = 10) were excluded, yielding a final sample of 2,999 children. Most of the mothers were born in France (94.6%), their age was 31.1 (*SD* 4.7) years on average, and 65.0% had attained an educational level of 2–3 years of university or more. The characteristics of the children are described in Table 1. Children were mainly first-born (77.1%) and balanced in gender (48.1% female).

**Table 1:** Characteristics of the participants.

	**Age class in months**														
	**All**^**a**^	**4-5**	**6**	**7**	**8**	**9**	**10**	**11**	**12**	**13-15**	**16-18**	**19-21**	**22-24**	**25-29**	**30-36**
	***N* = 2,999**	***N* = 142**	***N* = 283**	***N* = 235**	***N* = 243**	***N* = 187**	***N* = 195**	***N* = 168**	***N* = 137**	***N* = 370**	***N* = 279**	***N* = 254**	***N* = 178**	***N* = 203**	***N* = 125**
**CHILDREN'S CHARACTERISTICS**															
**Current weight-for-length z-score [mean (sd)]**	0.16 (1.2)	−0.01 (1.3)	0.04 (1.3)	0.07 (1.7)	0.16 (1.4)	0.15 (1.3)	0.28 (1.1)	0.28 (1.1)	0.10 (1.4)	0.32 (1.1)	0.29 (1.3)	0.31 (1.3)	0.20 (1.1)	−0.05 (1.0)	−0.15 (1.3)
**Number of teeth [mean (sd)]**	6.5 (6.3)	0.5 (1.9)	0.4 (1.3)	0.7 (1.5)	1.4 (2.0)	2.4 (2.2)	3.3 (2.5)	3.9 (2.3)	5.4 (2.5)	7.0 (2.9)	10.3 (3.6)	13.4 (3.6)	15.2 (3.1)	16.4 (2.7)	18.2 (2.4)
**Girls [*****N*** (%) or %]^*^	1,442 (48.1)	51.4	47.0	48.1	48.5	51.3	46.7	42.9	43.8	49.5	50.2	50.0	48.3	49.3	40.0
**Birth order [*****N*** **(%) or %]**															
1st born	2,290 (77.1)	74.3	84.3	75.1	76.2	72.7	78.2	77.7	74.6	77.1	76.0	73.0	79.1	79.2	81.2
**CHILDREN FEEDING SKILLS**															
**Sitting alone [*****N*** **(%) or %]**															
Sometimes/often	2,599 (86.8)	29.8	42.9	66.9	83.9	93.6	99.5	97.6	100.0	99.7	100.0	100.0	100.0	100.0	100.0
Never/rarely	394 (13.2)	70.2	57.1	33.1	16.1	6.4	0.5	2.4	0.0	0.3	0.0	0.0	0.0	0.0	0.0
**Drooling [*****N*** **(%) or %]**															
Sometimes/often	1,997 (66.6)	96.5	97.2	91.1	92.6	84.0	80.0	81.6	78.8	58.3	53.6	37.4	38.2	23.7	10.5
Rarely/never	999 (33.31)	3.5	2.8	8.9	7.4	16.0	20.0	18.5	21.2	41.7	46.4	62.6	61.8	76.4	89.5
**Pacifier using [*****N*** **(%) or %]**															
Often	1,451 (48.4)	51.4	47.4	52.8	49.8	55.6	49.7	45.8	48.2	47.7	46.6	48.0	53.4	36.5	46.4
Sometimes/rarely	679 (22.6)	26.1	28.3	22.6	24.3	21.9	24.1	26.2	18.3	25.5	20.4	16.5	19.1	20.7	19.2
Never	868 (28.9)	22.5	24.4	24.7	25.9	22.5	26.2	28.0	33.6	26.8	33.0	35.4	27.5	42.9	34.4
**Thumb sucking [*****N*** **(%) or %]**															
Sometimes/often	908 (30.3)	62.7	58.0	52.8	50.6	34.8	26.7	26.8	20.4	17.3	16.9	15.8	8.5	19.3	10.5
Rarely/never	2,088 (69.6)	37.3	42.1	47.2	49.4	65.2	73.3	73.2	79.6	82.7	83.2	84.3	91.5	80.7	89.5
**Eating with fingers [*****N*** **(%) or %]**															
Sometimes/often	1,605 (54.0)	7.2	7.2	10.5	16.6	29.7	43.3	46.7	69.4	74.5	87.8	90.6	88.8	91.6	86.4
Rarely/never	1,368 (46.0)	92.8	92.8	89.5	83.4	70.3	56.7	53.3	30.6	25.5	12.2	9.4	11.2	8.4	13.6
**Self-feeding with a fork [*****N*** **(%) or %]**															
Sometimes/often	827 (28.0)	1.4	0.0	0.0	0.4	0.6	1.1	3.6	1.5	12.9	45.4	70.1	83.7	96.1	97.6
Rarely/never	2,110 (72.0)	98.6	100.0	100.0	99.6	99.4	98.9	96.4	98.5	87.1	54.6	29.9	16.3	3.9	2.4
**Gagging [*****N*** **(%) or %]**															
Sometimes/Often	645 (21.8)	27.5	30.9	29.7	32.1	27.4	19.5	24.7	24.6	18.9	17.1	13.4	14.9	9.9	14.0
Rarely	1,050 (35.6)	31.9	32.6	42.7	38.0	37.1	39.5	36.8	37.3	42.7	31.3	35.2	35.1	24.4	24.0
Never	1,258 (42.6)	40.6	36.5	27.6	29.9	35.5	41.0	38.5	38.1	38.4	51.6	51.4	50.0	65.7	62.0
**FEEDING PRACTICES**															
**Any breastfeeding [*****N*** **(%) or %]**	1,886 (62.9)	55.6	62.9	63.8	67.1	63.6	63.1	62.5	63.5	56.5	63.4	65.0	67.4	64.0	64.8
**Age of CF [mean (sd)]**	4.9 (1.1)	4.1 (0.4)	4.4 (0.6)	4.7 (0.7)	4.8 (0.8)	4.9 (0.9)	4.9 (0.8)	4.8 (0.8)	4.9 (1.2)	5.0 (1.0)	5.1 (1.2)	5.0 (1.1)	5.0 (1.1)	5.2 (1.7)	5.1 (1.7)
**Meal taken with the family [*****N*** (%) or %]^*^	1,227 (40.9)	19.7	14.1	22.6	26.8	26.2	21.0	27.4	33.6	41.1	54.1	59.8	71.4	82.3	88.0
**Attendance to day care meal [*****N*** (%) or %]^*^	951 (32.0)	18.6	26.4	26.4	28.8	27.8	24.7	30.5	27.7	33.0	41.9	41.1	36.0	37.8	41.0
**BLW knowledge [*****N*** (%) or %]^*^	289 (9.7)	4.2	8.0	14.3	10.8	11.9	8.5	9.4	8.6	9.2	10.2	8.0	9.4	10.6	11.7
**BLW application [*****N*** (%) or %]^*^	55 (1.8)	0.0	0.0	2.5	1.6	1.1	3.1	1.8	0.7	2.4	3.6	1.6	1.7	1.5	2.4
**FOOD PREPARATION TYPES [*****N*** **(%) OR %]**															
Exclusive use of ready-prepared baby foods	396 (13.2)	22.5	21.2	20.9	16.5	19.3	18.5	19.6	14.6	13.5	5.4	5.5	3.4	2.0	0.8
Use of both ready-prepared baby foods and homemade foods	1,588 (53.0)	54.2	58.0	62.1	61.3	65.8	59.5	58.9	67.2	58.1	50.5	50.8	37.1	26.1	14.4
Exclusive use of homemade food and/or non-specific foods	1,015 (33.8)	23.3	20.8	17.0	22.2	15.0	22.0	21.4	18.2	28.4	44.1	43.7	59.6	71.9	84.8
**FEELINGS REGARDING THE INTRODUCTION OF SOLIDS [*****N*** **(%) OR %]**															
Unconcerned	1,225 (40.9)	37.3	41.0	43.4	42.8	41.1	34.4	36.9	35.7	38.1	42.6	40.2	43.3	43.8	53.6
Eager	793 (26.4)	50.0	41.3	36.6	36.6	20.9	25.6	28.0	25.6	19.5	16.1	20.9	20.2	13.8	20.0
Reluctant	981 (32.7)	12.7	17.7	20.0	20.6	38.0	40.0	35.1	38.7	42.4	41.2	39.0	36.5	42.4	26.4

*CF, complementary feeding; BLW, baby-led weaning*.

* *Dichotomous variables: Only the “yes” modality is presented; for the sex variable, only the girl modality is presented*.

a *For some age classes and some variables, information was not reported, and the total number of responses is not always equal to 2,999*.

Children's motor skills evolved as a function of age (Table 1). Most of the children (>80%) were reported to be able to sit alone at 8 months, to eat with their fingers at 16–18 months and with a fork at 22–24 months (Table 1). The proportion of children having “sometimes/often” gag reflex was ~30% in 6-to-8-month-old children and decreased to <15% in children aged 19–21 months and older. The frequency of thumb sucking decreased with age (62.7% of 4–6-month-old to 10.5% of 30–36-month-old children), whereas frequent pacifier use was relatively constant across ages (48.4% on average). The number of teeth increased with age (from 0.5 ± 1.9 at 4–5 months to 18.2 ± 2.4 at 30–36 months). For maternal feeding practices, children were on average introduced to CF at 4.9 months, and 62.9% of them were/had been breastfed. At 12 months, the frequency of children taking part in family meals was 33.6%; it then increased to 88.0% in 30–36-month-old children. Mothers very rarely used the baby-led weaning (BLW) method (1.8%). Mothers were mainly feeding their child by using both commercial baby and homemade foods (53%). Exclusive use of ready-prepared baby foods decreased from 22.5% in 4- to 5-month-old children to <10.0% after 15 months. Most mothers were either unconcerned (40.9%) or eager (26.4%) with regards to the introduction of food pieces, whereas 32.7% were reluctant to introduce them.

### Pattern of Ability to Eat Different Textures as Function of Age

**Figure 1:**
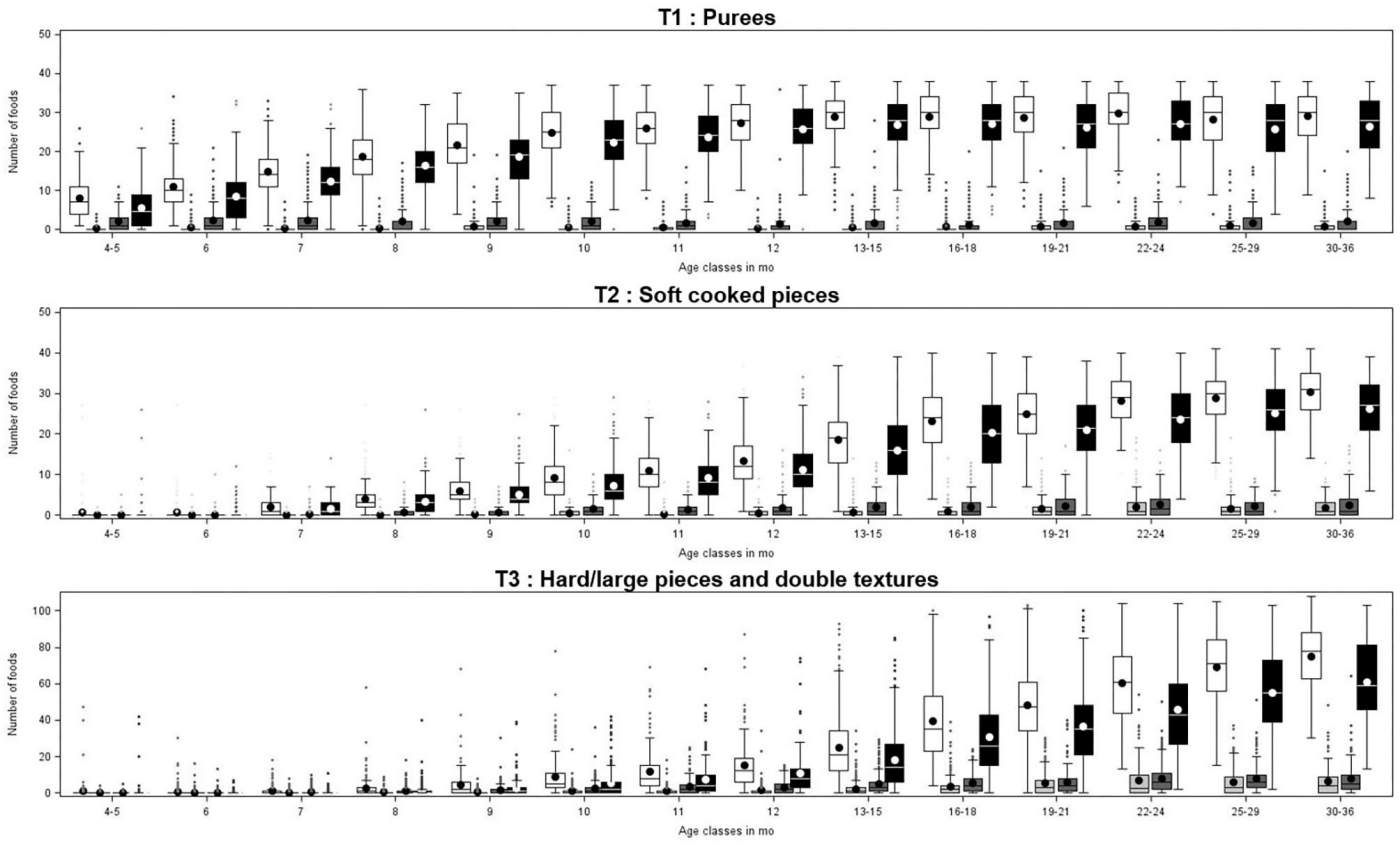
Mean number of food-texture combinations offered (white box) and their level of acceptance (spat out: light gray box, eaten with difficulties: dark gray box, and eaten without difficulty: black box) as a function of children's age class and texture levels (T1, T2, and T3).

**Figure 2:**
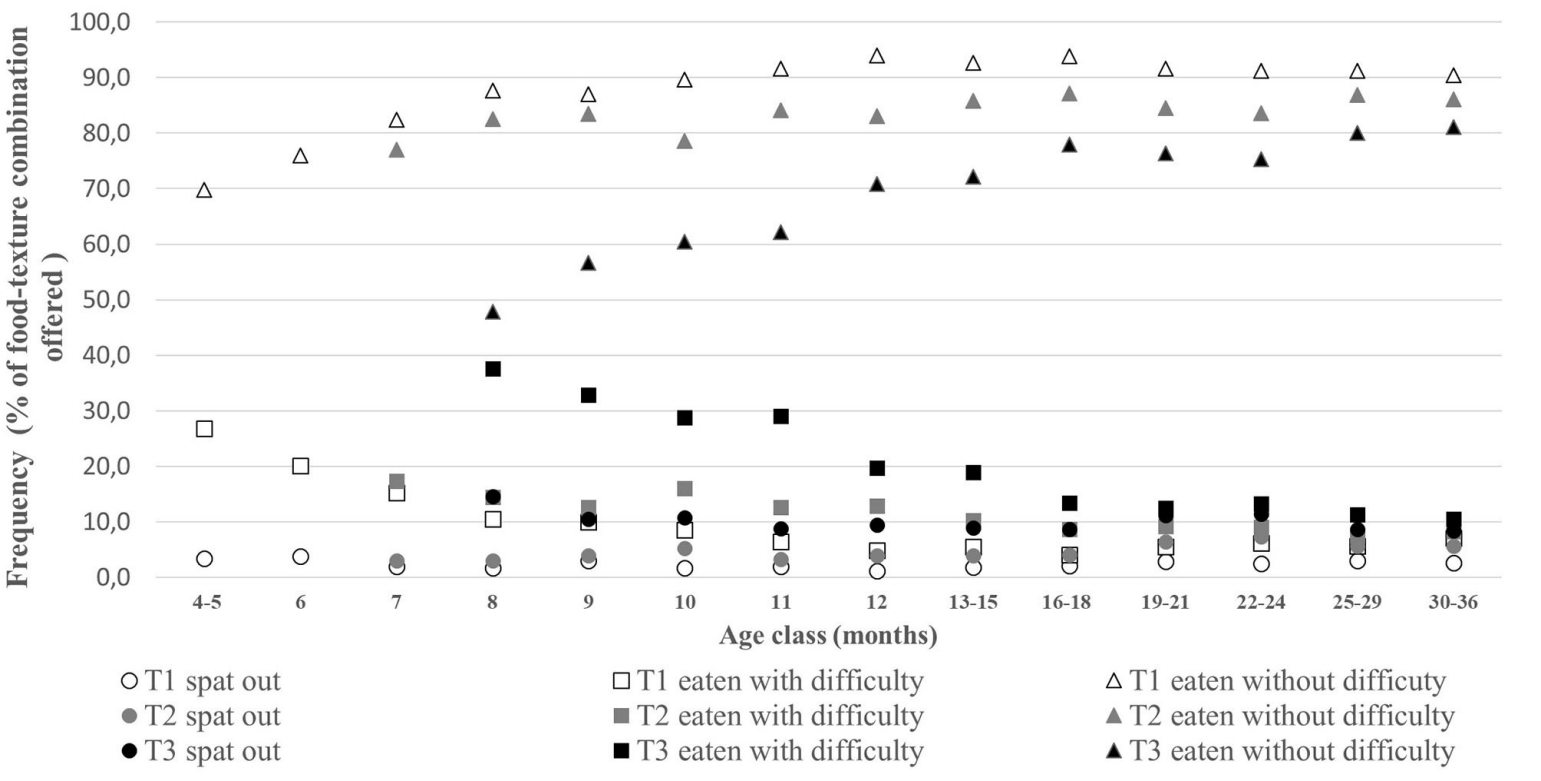
Mean percentages of the number of combinations “spat-out,” “eaten with difficulties,” and “eaten without difficulty” over the number of combinations offered, by texture level and by age class. Frequencies are presented when the median of offered combination(s) was at least equal to 1 (T2 and T3 data are thus missing up to 7 and 8 months, respectively).

The mean number of food-texture combinations “offered” and their level of acceptance are presented in Figure 1, and the ratios of the number of food texture combinations accepted vs. offered in Figure 2. Acceptance (i.e., ability to eat without difficulty) increased with age and was very much related to the offering pattern (Figure 1). Acceptance for soft and rough purees (T1 level) significantly increased with age [*F*_(13, 2982)_ = 36.9, *p* < 0.001]: it increased from 4/5 months to 7 months (from 70 to 82% of offered T1 combinations) and was relatively stable afterwards (between 87 and 93% in the period from 8 to 30–36 months old) (Figure 2). The proportion of small and soft pieces (T2 level) eaten without difficulty is 5 to 10% lower than that of T1 level items. The age effect for the acceptance of small and soft pieces was smaller but still significant [*F*_(11, 2437)_ = 2.2, *p* = 0.01; with significant differences between 10-month-old children and those aged between 25 and 36 months]. For the more complex textures (T3 level), the acceptance vs. offered ratio steadily increased with age [*F*_(10, 2153)_ = 29.4, *p* < 0.001], and the age classes between 8 and 11 months had a significantly lower proportion of accepted foods (47–62%) than the older age classes (>70%). Within the older age classes, T3 level acceptance significantly increased between 12–13/15 months (~71%) and 25/29–30/36 months (~80%).

The increase in texture acceptance with age was associated with a decrease in the difficulties in eating (*p* < 0.05 for all texture levels). The ratio of foods directly spat out vs. food texture combinations offered also decreased with age (*p* < 0.05 for all texture levels) and concerned relatively few foods in all age classes: 2.5% of the T1 foods, 4.5% of the T2 foods, and 10% of the T3 foods. At all age classes, the ratio of food eaten without difficulty vs. foods offered ranked in the following order: T1 > T2 > T3. These differences were significant (*p* < 0.001), except for the age classes 7, 8, and 9 months, where the ratios for T1 and T2 were not significantly different.

### The Texture Acceptance Score (TextAcc) as a Function of Age

**Figure 3:**
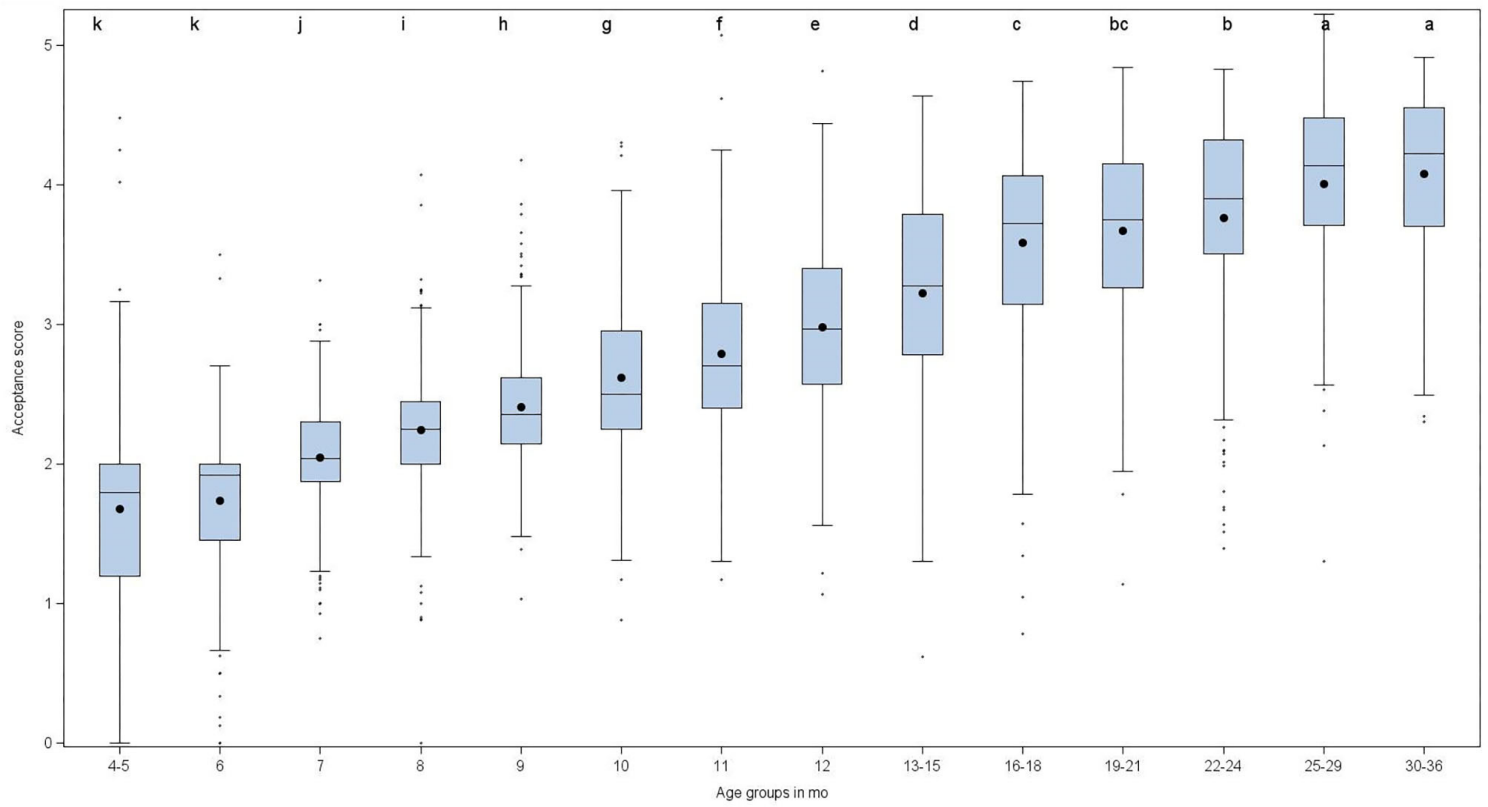
Texture acceptance score (TextAcc) per age class. Mean scores associated with different letters (a, b, …, k) are significantly different (*p* < 0.05).

The distribution of the TextAcc over age classes is presented in Figure 3. As expected, TextAcc significantly increased with age [*F*_(13, 2985)_ = 381.7, *p* < 0.0001]. The score increased steadily between 4–5 and 16–18 months (1.7 ± 0.7 to 3.6 ± 0.7, respectively) and then more slowly up to 30–36 months (4.1 ± 0.6) (Figure 3). This shows that new food texture combinations become accepted throughout the entire CF period. The high variability of this score raises the question of its main predictors.

### Factors of Food Texture Acceptance

Among the 17 variables initially associated with TextAcc (Supplementary Material 3), eight were still significant when assessed in multivariate analysis corrected for weight for length z-score (Table 2). TexAcc was better explained for children in the age classes between 9 and 16–18 months, as observed from the *R*^2^ (Table 2), than for those of earlier or later ages.

**Table 2:** Associations between food texture acceptance score (TextAcc) and children's characteristics and skills and maternal feeding practices from multiple linear regression models performed by age class [the reported figures are beta values (95% confidence intervals)].

**Age groups (in months)**	**Tested variable modality**	**4-5**	**6**	**7**	**8**	**9**	**10**	**11**	**12**	**13-15**	**16-18**	**19-21**	**22-24**	**25-29**	**30-36**
***N***		**142**	**283**	**235**	**243**	**187**	**195**	**168**	**137**	**370**	**279**	**254**	**178**	**203**	**125**
***N*** **observations used**		**128**	**269**	**222**	**231**	**174**	**190**	**156**	**127**	**351**	**255**	**256**	**150**	**163**	**88**
**EXPOSURE TO FOOD TEXTURE**															
T1		0.03^*^	0.00	−0.00	−0.01	−0.02^***^	−0.02^***^	−0.01^**^	−0.02^**^	−0.02^***^	−0.04^***^	−0.02^*^	−0.02	−0.03^***^	−0.05^***^
		**(0.00, 0.05)**	(−0.01, 0.02)	(−0.02, 0.01)	(−0.02, 0.00)	**(−0.03**, **−0.01)**	**(−0.03**, **−0.01)**	**(−0.03**, **−0.06)**	**(−0.04**, **−0.01)**	**(−0.03**, **−0.01)**	**(−0.05**, **−0.03)**	**(−0.03**, **−0.00)**	(−0.05,0.01)	**(−0.05**, **−0.02)**	**(−0.07**, **−0.02)**
T2		–	0.08^**^	0.07^***^	0.05^***^	0.06^***^	0.04^***^	0.04^***^	0.01	0.03^***^	0.04^***^	0.01	0.01	0.00	0.02
			**(0.03, 0.14)**	**(0.04, 0.10)**	**(0.02, 0.07)**	**(0.04, 0.08)**	**(0.02,0.05)**	**(0.02, 0.05)**	(−0.01, 0.03)	**(0.02, 0.05)**	**(0.02, 0.06)**	(−0.01, 0.02)	(−0.03, 0.04)	(−0.02, 0.03)	(−0.02, 0.07)
T3		–	0.04^*^	0.03^*^	0.03^***^	0.02^**^>	0.02^***^	0.02^***^	0.02^***^	0.03^***^	0.01^**^	0.01^***^	0.01^**^	0.02^***^	0.01
			**(0.01, 0.08)**	**(0.01, 0.06)**	**(0.01, 0.04)**	**(0.01, 0.03)**	**(0.02, 0.03)**	**(0.01, 0.03)**	**(0.01, 0.03)**	**(0.02, 0.05)**	**(0.00, 0.01)**	**(0.01,0.02)**	**(0.01, 0.03)**	**(0.01, 0.03)**	(−0.01, 0.02)
**CHILDREN CHARACTERISTICS AND SKILLS (MODALITY OF REFERENCE)**															
Number of teeth		0.16^***^	0.02	−0.01	0.00	0.02	0.03^*^	0.02	0.03	0.02^*^	0.00	0.00	−0.02	0.01	0.02
		**(0.09; 0.23)**	(−0.06, 0.10)	(−0.04, 0.03)	(−0.03, 0.03)	(−0.00,0.46)	**(0.00, 0.05)**	(−0;01, 0.05)	(−0.05, 0.06)	**(0.00, 0.04)**	(−0.01, 0.02)	(−0.02, 0.02)	(−0.05, 0.02)	(−0.02, 0.04)	(−0.03, 0.06)
Current weight–for–length z–score		0.00	−0.02	0.04	0.04^*^	0.03	0.00	−0.00	0.05	−0.02	0.04	0.04	0.00	0.02	−0.08
		(−0.07, 0.08)	(−0.06,0.03)	(−0.01, 0.08)	**(0.00, 0.08)**	(−0.01, 0;07)	(−0.05, 0.06)	(−0.06, 0.05)	(−0.01, 0.11)	(−0.07, 0.02)	(−0.01,0;08)	(−0.01; 0.10)	(−0.10, 0.10)	(−0.05, 0.10)	(−0.17, 0.01)
Eating with fingers (sometimes/often)	Rarely/never	−0.05	−0.13	0.25^**^	−0.07	−0.06	−0.12	−0.21^***^	−0.24^*^	−0.13^*^	−0.31^**^	−0.32^*^	−0.04	−0.02	0.15
		(−0.49, 0.39)	(−0.36,0.11)	**(0.07, 0.42)**	(−0.21, 0.07)	(−0.19, 0.06)	(−0.25, 0.01)	**(−0.34**, **−0.09)**	**(−0.45**, **−0.04)**	**(−0.25**, **−0.01)**	**(−0.49**, **−0.11)**	**(−0.58**, **−0.07)**	(−0.38, 0.30)	(−0.31, 0.28)	(−0.19, 0.48)
Gagging frequency (never)	Rarely	−0.19	−0.16^*^	−0.02	−0.05	−0.06	−0.02	−0.16^*^	−0.07	−0.08	−0.06	−0.17^*^	0.07	−0.06	−0.26
		(−0.43, 0.13)	**(−0.30**, **−0.02)**	(−0.14, 0.10)	(−0.17, 0.07)	(−0.18, 0.06)	(−0.15, 0.12)	**(−0.29**, **−0.01)**	(−0.27, 0.12)	(−0.20, 0.02)	(−0.19, 0.08)	**(−0.32**, **−0.02)**	(−0.18, 0.31)	(−0.23, 0.11)	(−0.54, 0.02)
	Sometimes/often	−0.16	−0.18^*^	−0.01	−0.02	−0.13	−0.14	−0.19^*^	−0.24^*^	−0.26^***^	−0.36^***^	−0.05	−0.27	−0.50^**^	−0.16
		(−0.40, 0.09)	**(−0.32**, **−0.04)**	(−0.15, 0.12)	(−0.15,0.10)	(−0.27, 0.00)	(−0.31, 0.03)	**(−0.34**, **−0.03)**	**(−0.47**, **−0.05)**	**(−0.40**, **−0.12)**	**(−0.50**, **−0.17)**	(−0.29, 0.16)	(−0.59, 0.06)	**(−0.80**, **−0.20)**	(−0.49, 0.18)
**FEEDING PRACTICES (MODALITY OF REFERENCE)**															
Age of CF		−0.15	−0.17^**^	−0.04	−0.05	−0.05	−0.02	−0.00	−0.10^**^	0.00	−0.03	0.02	0.05	−0.02	0.02
		(−0.44, 0.13)	**(−0.29**, **−0.06)**	(−0.11, 0.03)	(−0.12,0.01)	(−0.10, 0.01)	(−0.09, 0.06)	(−0.09,0.07)	**(−0.17**, **−0.03)**	(−0.05, 0.05)	(−0.08, 0.02)	(−0.05, 0.08)	(−0.07, 0.16)	(−0.08, 0.03)	(−0.05, 0.08)
Feelings re: introduction of solids (unconcerned)	Eager	0.02	0.07	−0.03	0.01	−0.06	0.12	−0.10	−0.03	−0.12	0.02	−0.17	−0.10	0.16	−0.24
		(−0.20, 0;23)	(−0.05, 0.20)	(−0.15, 0.08)	(−0.10, 0.12)	(−0.21, 0.09)	(−0.04, 0.28)	(−0.25, 0.05)	(−0.28, 0.18)	(−0.24, 0.03)	(−0.16, 0.19)	(−0.36, 0.02)	(−0.42, 0.21)	(−0.07, 0.39)	(−0.54, 0.06)
	Reluctant	0.00	0.05	−0.15^*^	−0.04	−0.13^*^	0.03	−0.19^*^	−0.00	−0.18^**^	−0.08	−0.16	−0.26	0.06	−0.31^*^
		(−0.32, 0.32)	(−0.13, 0.22)	**(−0.29**, **−0.02)**	(−0.17, 0.10)	**(−0.25**, **−0.01)**	(−0.11,0.18)	**(−0.34**, **−0.03)**	(−0.20, 0.20)	**(−0.29**, **−0.07)**	(−0.21, 0.06)	(−0.33, 0.00)	(−0.54, 0.03)	(−0.11, 0.23)	**(−0.61**, **−0.02)**
**Model** ***R**^**2**^*		0.31	0.20	0.29	0.36	0.52	0.52	0.62	0.48	0.59	0.56	0.34	0.27	0.48	0.37

*Significant effects are highlighted in bold*:

* p < 0.05,

** p < 0.01,

*** p < 0.001).

For categorical data, the modality of reference is specified within brackets.

*“–:” not tested because mostly not offered in this age class*.

*CF, complementary feeding*.

At most age classes, TexAcc was related to the numbers of food-texture combinations offered (T1, T2, and T3; Table 2). The direction of the association depended on the texture level considered. TextAcc was positively associated with the number of T2 foods introduced in the age class between 6 and 16–18 months (except at 12 months, *p* = 0.32) and with the number of T3 foods for the classes from 6 months up to 25–29 months. TextAcc was negatively associated with the number of T1 foods introduced in the age classes between 9 and 30–36 months [except for the 22–24 months class (*p* = 0.12)]. In other words, the less children were exposed to purees and the more they were exposed to pieces (soft or hard), the higher their acceptance score.

Other factors that were associated with TextAcc included some children's developmental characteristics. The TextAcc score was mainly related to the ability to eat with fingers. Among 11-, 12-, 13–15-, 16–18-, and 19–21-month-old children, those who never/rarely ate with their fingers had lower acceptance scores than those doing so more frequently (Table 2). Surprisingly, a significant opposite effect was observed at 7 months, an age period when food pieces (T2 and T3) were barely introduced. TextAcc was also related to gagging (Table 2). In the following age classes, 6, 11, 12, 13–15, 16–18, 19–21, and 25–29 months, children reported to rarely or often gag had a lower TextAcc score than those for whom this behavior was never observed (Table 2). The number of teeth was associated with a higher TextAcc score for the group of 4-5-, 10-, and 13–15-month-old children.

Concerning feeding practices, 6- and 12-month-old children introduced earlier to CF had a higher texture acceptance score. Finally, the feeling reported by mothers concerning the introduction of solids was significantly associated with TextAcc. For 7, 9, 11, 13–15, and 30–36-month-old children, the children of mothers who reported themselves as being reluctant to introduce solids had a lower texture acceptance score than those of mothers who were unconcerned.

## Discussion

This study aimed to evaluate parental self-reports of children's ability to eat foods of different textures and to determine factors associated with children's texture acceptance as a function of age. Texture Acceptance (proportion of foods texture-combination easily eaten over the total introduced) increased from the beginning of CF until the end of the 3rd year of life and decreased with texture level (purees > soft and small pieces> big/hard pieces and double texture) at each age studied. Associated factors were related to specific aspects of parental feeding practices and feelings concerning food piece introduction and some developmental characteristics of children.

### Food Texture Acceptance: Evolution With Age

Patterns of texture acceptance (i.e., ability to eat without difficulty) were closely related to the patterns of food offering, suggesting that when parents offered solid foods with a specific texture to their child, these foods or textures generally became accepted without difficulty. This could be explained by the fact that food textures are introduced in the diet in a period when children have already acquired the necessary skills to eat them or can easily develop them upon exposure to textures. This is in agreement with the previous observation that non-pureed food-texture combinations (T2 and T3) were introduced rather late to children in France [see also (31)] and that children were able to handle textures in small quantities at an earlier age than their parents' feeding practices (14).

Acceptance developed mainly between the start of CF up to 7 months for pureed foods, which is in agreement with the acceptance frequency for smooth and rough purees observed at 6 months (14). Acceptance for small and soft pieces and after that, more challenging textures (T3 levels) develop up to 30–36 months in our study. The increase in acceptance is related to the transition from sucking to chewing [~8–10 months, (14)], the development of chewing skills for textured foods during the CF period, as observed earlier from the number of chews required to swallow foods (4) and the ability to form particles from a model food gel (13). An earlier study based on a parental report conducted in the US (28) reported that children were eating food with tiny lumps without gagging at 8.7 months (age range: 4.8–15.5) and chewed softer foods at 9.4 months (6.0–14.0). At this age (~9 months), we observed that 87% of the purees (both smooth and rough) and 83% of small/soft pieces (that can be squeezed between the tongue and palate) were eaten without difficulty. Carruth and Skinner (28) reported that children are able to chew and swallow firmer foods without choking at ~12.2 months, although with a very large age range (7.5 and 20.0 months). In our study, acceptance for T3 texture was ~70% at 12 months and was found to continue to develop until 30/36 months. This development is in line with the development of chewing function characterized by mandibular motor control and chewing muscle coordination (11). Despite an increase in texture acceptance with age throughout the entire CF period and a decrease in the gap between texture levels, we observed that purees were still on average better accepted (eaten without difficulty) than soft and small pieces and that hard/large pieces and double texture foods were the least easily eaten at the end of the CF period.

### Acceptance and Maternal Feeding Practices

Food texture introduction was found to be the main factor associated with acceptance. In a given age group, children having the higher acceptance score were those who had been offered the opportunity to experience a large variety of foods offered as pieces or double texture (soft and small pieces until 18 months and hard/large pieces and double texture until 29 months). This is in agreement with two previous studies, which concluded that the age of introduction of lumpy foods (17, 18) and familiarity with different textures (15) are important factors for developing food acceptance. All three studies contribute to the notion that a timely and repeated introduction of a variety of textured food is needed to achieve good food acceptance.

Concerning other maternal feeding practices evaluated in the survey, children in the present study were introduced to CF (timing and type of food) in agreement with the National French guidelines (34), [see (31) for discussion on practices] and mostly via traditional spoon feeding. Some factors were initially associated with TexAcc (breastfeeding in children of 10, 12, 20/24 months, eating with the family (10 of 14 age classes), exclusive use of homemade or non-baby commercial foods (eight age classes) and exclusive use of ready-prepared baby foods (11, 13/15, and 16/18 months) but were no longer significant when assessed in multivariate analysis, suggesting that they may play an indirect role in acceptance by influencing maternal practices with regard to texture introduction.

Last, compared to mothers who were reluctant to introduce solid foods, mothers who were unconcerned about the introduction of solids had children who better accepted texture. This is partly in agreement with the earlier observation that reluctant mothers introduced less texture in their children's diet than other mothers (31). As texture introduction was taken into account in the current analysis, data suggest that maternal feelings concerning foods for their children may also affect measured acceptance *via* an additional way than limited exposure to textured foods. This way can be 2-fold: mothers reluctant to introduce solids may have underestimated their child's acceptance of texture, or they may have insisted less when proposing a food with a difficult texture during the meal. It would be interesting in future studies to better understand the reluctance of some mothers to introduce foods pieces, as this may help to find ways to improve texture acceptance in their child.

### Acceptance of Food Textures and Associations With Developmental Characteristics of Children

Reported motor and feeding skills evolved with age and were congruent with the time line of typically developing children reported previously: ability to sit alone (28) and drooling frequencies (35) were in agreement with previous reports. Approximately half of infants were able to eat with their fingers at 11–12 months, which seems later than reported in a US survey where 98% of 9-11-month-old children were reported to grasp foods with their hand (36). Gagging frequency was reported sometimes/often in 20–30% of the children between 4 and 12 months, which is within the frequency range observed from video analysis of 8- to 9-month-old children eating pieces (19). Gagging and eating with fingers predicted acceptance for children in the age classes between 11 and 29 months. Children self-feeding with their fingers frequently accepted textured foods better than those doing it less often. The beginning of the 2nd year coincides with the introduction of soft and hard textures. At this stage, the child's tactile experience is stimulated at both the digital and oral levels: first, he/she holds the food with his/her hands, and then, he/she continues exploring it with the mouth (37). The present study is in agreement with others run in preschool and school children reporting that feeling the texture with their hands increased acceptance of a food with the same texture (23, 38). Gagging was associated with lower texture acceptance at the beginning of CF (6 months) and later during CF (11, 12, 13–15, 16–18, and 25–29 months). These ages correspond to the introduction of the first non-smooth purees (i.e., rough purees at 6 months) and of foods with pieces and harder texture (2nd year of life). A relationship between gagging and texture has been reported earlier in 8-month-old children: gagging in response to food pieces was more frequent than in response to pureed foods (19). Gagging is a normal reflex due to the high tactile sensitivity of the inner sides of the cheeks (39) and usually decreases upon repeated exposure to (new) textures. However, gagging has been reported earlier as behavior occurring in children presenting eating difficulties (40) and in tactile defensive children (22). Here, gagging was evaluated as a general behavior of the child and not as a specific behavior related to a given food, so unfortunately, these data do not enable a further understanding of which food texture may specifically provoke a gag response.

Texture acceptance was also initially associated with children's ability to sit alone in young children (in the age classes between 4–5 and 7 months) and with the ability to eat alone with a fork in the older children (classes 13–15 to 24 months). However, these skills were no longer associated in the multivariate model, suggesting that they indirectly impact food texture acceptance by influencing food texture exposure.

For few age classes, texture acceptance was associated with children's dentition. The number of teeth reported by parents was in agreement with normal dental eruption (41, 42) and was significantly associated with texture acceptance for 4- to 5-, 10-, and 13- to 15-month-old children, suggesting the favorable role of incisors and first molars (which are known to erupt around this age) on children's ability to eat solids. Initial binary analyses (Supplementary Material 3) revealed a positive association between dentition and texture acceptance (*p* < 0.05) for six age classes (4-5, 8, and between 10 and 15 months). After multivariate analyses, this effect remained significant for only three of them, suggesting that the associations between dentition and acceptance were confounded with other factors having more impact on acceptance. Indeed, we previously reported that dentition is a predictive factor for exposure to texture: the number of teeth was considered by mothers as a signal for introducing textured food (31).

### Strengths and Limitations

Using parental reports, details of children's texture acceptance have been generated during the entire CF period for a wide range of food textures and based on a relatively large number of children. Moreover, the extent to which individual characteristics, children's feeding skills and other personal characteristics as well as maternal feeding practices and feelings influence acceptance was evaluated. Our results are in agreement with previous experimental studies concerning both the development of texture acceptance and the favoring factors, which suggests that parental reports of children's eating ability are a valuable assessment that could be used in future studies. The factors associated with texture acceptance were hierarchized between those directly affecting acceptance and those influencing exposure.

However, some limitations to this work are worth mentioning. The included population is a convenient sample, which limits the generalization of the results to the national population. Primiparity and educational attainment were higher in our sample than reported in the general French population (43). These parameters are known to influence maternal feeding practices and therefore may have impacted the results. In addition, participating mothers were registered and recruited *via* the Bledina potential consumers database; however, they were not necessarily feeding their child (exclusively) with commercial baby food (only 14% of them were exclusively using this type of food to feed their child). Thus, future works should aim to extend the current study in a sample that is more representative of the national population, in terms of both parents' and children's characteristics.

A second limitation concerns the design of the questionnaire. First, in its present format, it contains 188 food-texture combinations, which is a compromise between covering a wide range of relevant foods and textures, trying to have a balanced list of foods and textures for the different food categories and being short enough for parents to not be discouraged from completing it. The results of our study suggest that the questionnaire could be simplified for future studies without losing information, as current data allowed us to identify a subset of combinations rarely offered to children regardless of age. Second, although a previous study from us revealed a good agreement between parents and experimenter evaluation when assessing acceptance based on swallowing a food in a behavioral situation [(14), data not shown], a validation of the parental responses in the survey against behavioral measures has not yet been done and cannot be made with the current data set. Ideally, a future study should evaluate the validity and reproducibility of this questionnaire. Finally, the reported relationships between some developmental characteristics of children—food exposure—food acceptance are correlational, and further research based on randomized interventions should be done to help us to better understand these relationships.

## Conclusion

The development of food texture acceptance during the complementary feeding period and associated factors were determined from parental reports of their child's ability to eat selected foods and textures. This study confirmed that complementary feeding is an important period for children to accept new textures. Acceptance develops with age upon exposure to a variety of textures, and in each age class, it varies as a function of the texture level. Some developmental characteristics of children, maternal feeding practices and maternal feelings with regard to the introduction of solids were associated with acceptance either directly or by modulating exposure. Children's ability to eat with their fingers, frequency of gagging and, to a lesser extent, their dentition and their mothers' feelings with regard to the introduction of solids were the major predictors of acceptance. This survey gives important information on the development of acceptance for textured foods by children over the entire complementary feeding period in France. It confirmed the importance of prior exposure to a variety of textures for the acceptance of textured foods and provides evidence regarding the involvement of several personal factors.

## Data Availability Statement

The datasets presented in this article are available by request to corresponding authors, after permission by the industrial partner. Requests to access the datasets should be directed to Carole Tournier, carole.tournier@inrae.fr.

## Ethics Statement

The studies involving human participants were reviewed and approved by Comité de Protection des Personnes Est III, no. 2015-A00323-46. The patients/participants provided their written informed consent to participate in this study.

## Author Contributions

CT, LD, AM, HW, and SN designed the research. LD collected the data. LD, CT, EK, and SN analyzed the data. CT and SN wrote the initial paper, which was then reviewed by LD, AM, and HW. All authors are responsible for the study findings, read, and approved the manuscript. All authors contributed to the article and approved the submitted version.

## Conflict of Interest

LD and AM are employees of Blédina R&I. HW has been an employee of Danone Nutricia Research. The remaining authors declare that the research was conducted in the absence of any commercial or financial relationships that could be construed as a potential conflict of interest.

## Abbreviations

TextAcc: Texture acceptance score
CF: complementary feeding.
