# Supplementary material for: Factors Associated With Food Texture Acceptance in 4- to 36-Month-Old French Children: Findings From a Survey Study
Source: Front Nutr. 2021 Feb 1;7:616484. doi: 10.3389/fnut.2020.616484 (PMC7882631; doi:10.3389/fnut.2020.616484)
Supplement: Supplementary file 1 [file Data_Sheet_1.PDF]

Supplementary material

Supplementary Table 1:

List of the 188 food-texture combinations and their texture levels assignment (presented in this order in the questionnaire)

Food category	Food-texture combination	Texture level
Commercial baby foods	Fruit purees	T1
	Yoghurt	T1
	Custard	T1
	Petit-Suisse cheese	T1
	Rice pudding	T1
	Ready-prepared infant cereals	T1
	Biscuits	T3
	Vegetable soup	T1
	Smooth vegetable purees	T1
	Rough vegetable purees	T1
	Preparation for 6 mo-old infants	T1
	Preparation for 8-10 mo-old infants	T1
	Preparation for 12 mo-old infants	T2
	Preparation for 15 mo-old infants	T2
	Preparation for 18 mo-old infants	T3
	Preparation for 24 mo-old infants	T3
Vegetable and potato	Smooth carrot puree	T1
	Rough carrot puree	T1
	Cooked carrot in small pieces	T2
	Cooked carrot in large pieces	T3
	Grated raw carrot	T3
	Raw carrot in small pieces	T3
	Raw carrot in large pieces	T3
	Smooth green bean puree	T1
	Rough green bean puree	T1
	Cooked green bean in small pieces	T2
	Cooked green bean in large pieces	T3
	Whole cooked green beans	T3
	Rough spinach/leek purees	T1
	Cooked spinach/leek coarsely chopped	T2
	Cooked spinach/leek coarsely in pieces	T3
	Smooth cauliflower/broccoli purees	T1
	Rough cauliflower/broccoli purees	T1
	Cooked cauliflower/broccoli in small pieces	T2
	Cooked cauliflower/broccoli in large pieces	T3
	Smooth potato puree	T1
	Rough potato puree	T1
	French fries	T3
	Cooked potatoes in small pieces	T2
	Cooked potatoes in large pieces	T3
	Cooked minced tomato	T1
	Cooked tomato in small pieces	T2
	Cooked tomato in large pieces	T3
	Raw tomato in small pieces	T3
	Raw tomato in large pieces	T3
	Whole raw tomato	T3

Food category	Food-texture combination	Texture level
	Rough lentils/white beans/corn/green pea puree	T1
	Whole cooked lentils/white beans/corn/green pea	T3
	Rough zucchini/eggplant/pepper/pumpkin purees	T1
	Cooked zucchini/ eggplant /pepper/pumpkin in small pieces	T2
	Cooked zucchini/ eggplant/pepper/pumpkin in large pieces	T3
	Grated raw cucumber	T3
	Raw cucumber in small pieces	T3
	Raw cucumber in large pieces	T3
	Raw radish slices	T3
	Whole raw radish	T3
	Smooth beetroot/turnip/celery/parsnip puree	T1
	Rough beetroot/turnip/celery/parsnip puree	T1
	Cooked beetroot/turnip/celery/parsnip in small pieces	T2
	Cooked beetroot/turnip/celery/parsnip in large pieces	T3
	Mushroom soup	T1
	Cooked mushrooms in small pieces	T2
	Whole cooked mushrooms	T3
	Raw mushrooms in small pieces	T3
	Whole raw mushrooms	T3
	Cooked asparagus in pieces	T3
	Raw salad/cabbages in pieces	T3
	Cooked cabbage/cardoon/chard in small pieces	T2
	Cooked cabbage/cardoon/chard in large pieces	T3
	Unblended vegetable soup with pieces	T3
Fruit	Smooth applesauce	T1
	Rough applesauce	T1
	Cooked apple in small pieces	T2
	Cooked apple in large pieces	T3
	Raw apple in small pieces	T3
	Raw apple in large pieces	T3
	Whole raw apple	T3
	Smooth pear sauce	T1
	Rough pear sauce	T1
	Cooked pear in small pieces	T2
	Cooked pear in large pieces	T3
	Raw pear in small pieces	T3
	Raw pear in large pieces	T3
	Whole raw pear	T3
	Smooth banana puree	T1
	Rough banana puree	T1
	Raw banana in small pieces	T2
	Raw banana in large pieces. slices included	T3
	Smooth apricot/peach/plum/cherry puree	T1
	Rough apricot/peach/plum/cherry puree	T1
	Cooked apricot/peach/plum/cherry in small pieces	T2
	Cooked apricot/peach/plum/cherry in large pieces	T3
	Raw apricot/peach/plum/cherry in small pieces	T3
	Raw apricot/peach/plum/cherry in large pieces	T3
	Whole raw apricot/peach/plum/cherry	T3
	Raw citruses in small pieces	T3
	Raw citruses in large pieces	T3
	Raw red berries in pieces	T3
	Whole raw red berries	T3

Food category	Food-texture combination	Texture level
	Raw melon/watermelon in small pieces	T3
	Raw melon/watermelon in large pieces	T3
	Grape in pieces	T3
	Whole grape	T3
	Raw kiwi in small pieces	T3
	Raw kiwi in large pieces	T3
	Raw mango in small pieces	T3
	Raw mango in large pieces	T3
	Raw pineapple in small pieces	T3
	Raw pineapple in large pieces	T3
	Rough rhubarb puree	T1
	Cooked rhubarb in pieces	T2
	Fruit-based salad	T3
Meat	Minced cooked ham	T2
	Cooked ham in small pieces	T3
	Cooked ham in large pieces	T3
	Minced meat (all types of meat)	T2
	Cooked meat in small pieces	T3
	Cooked meat in large pieces	T3
	Cooked sausage in small pieces	T3
	Cooked sausage in large pieces	T3
	Cooked nuggets in small pieces	T3
	Cooked nuggets in large pieces	T3
	Whole cooked nuggets	T3
	Dry sausage in small pieces	T3
	Dry sausage in slices	T3
Fish	Breaded fish in small pieces	T3
	Whole breaded fish stick	T3
	Smoked salmon in small pieces	T2
	Smoked salmon in large pieces	T3
	Minced cooked fish (all types of fish)	T2
	Cooked fish in small pieces	T2
	Cooked fish in large pieces	T3
	Flaked canned tuna	T2
	Canned tuna in pieces	T3
	Shrimp in small pieces	T2
	Shrimp in large pieces	T3
	Whole shrimp	T3
	Surimi in small pieces	T2
	Surimi in large pieces	T3
	Whole surimi	T3
Egg	Hard-boiled egg	T2
	Fried egg	T2
	Omelette	T2
Cereals	Soft bread	T2
	Bread in small pieces	T3
	Slice of bread	T3
	Crusty bread ending	T3
	Bread crust	T3
	Small pasta (star pasta. letter pasta)	T2
	Shell-shape pasta	T2
	Long pasta in pieces (spaghetti. fettucini etc.)	T3

Food category	Food-texture combination	Texture level
	Other pasta (penne. farfalle)	T3
	Raviolis	T3
	Polenta. semolina. rice	T2
	Savoury cereal based preparations (i.e. risotto)	T2
	Sweet cereal based preparations (i.e. rice pudding)	T2
	Corn flakes	T3
	Puffed rice	T3
	Muesli	T3
	Porridge	T3
Cheese and dairy products	Hard cheese in small pieces	T3
	Hard cheese in stick	T3
	Cheese spread (e.g. kiri®)	T2
	Cheese spread in small pieces	T2
	Whole cheese spread	T3
	Soft cheese in small pieces	T2
	Slice of soft cheese	T3
	White cheese	T1
	White cheese with pieces	T3
	Yoghurt	T1
	Yoghurt with pieces	T3
	Petit-suisse cheese	T1
	Custard	T1
Miscellaneous savoury and sweet foods	Meringue	T3
	Madeleine	T3
	Wafer	T3
	Boudoirs	T3
	Butter-biscuit	T3
	Shortbread	T3
	Chocolate candy	T3
	Soft caramel	T3
	Jelly candy	T3
	Chocolate squares	T3
	Croissant	T2
	Milk bread	T2
	Chips	T3
	Little puff	T2
	Pretzel	T3

Suppl. Mat 2: Some examples of pictures illustrating the size of the pieces with a scale

'In order to help you filling the survey, pieces' size are illustrated using photographs' :

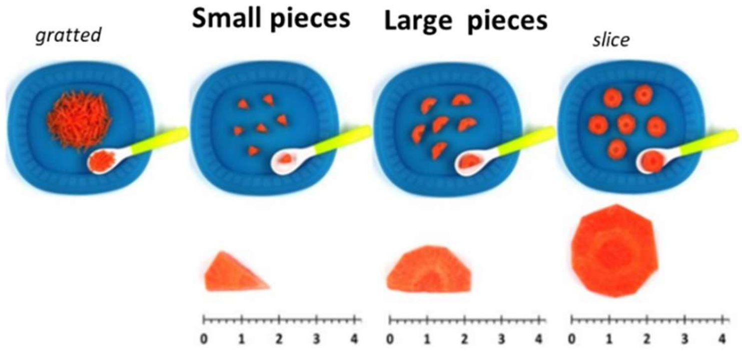

Supplementary Table 3: Associations between food texture acceptance score (TextAcc) and child characteristics, motor and feeding skills, maternal feeding practices and 'maternal feeling with regard to the introduction of solids' by age class (from 4 to 11 mo) from bivariate analysis. Values are standardized Betas, Significant effect ($p<0.05$) are highlighted in bold. CF: complementary feeding

**Age groups (in months)**	**Tested variable modality (for categorical characteristics)**	**4-5**	**6**	**7**	**8**	**9**	**10**	**11**	**12**	**13-15**	**16-18**	**19-21**	**22-24**	**25-29**	**30-36**
**n**		**142**	**283**	**235**	**243**	**187**	**195**	**168**	**137**	**370**	**279**	**254**	**178**	**203**	**125**
**Children characteristics (modality of reference)**															
Sex (boys)	Girls	0.01	-0.09	0.08	-0.04	-0.05	-0.04	**-0.19**<br>**	0.05	0.02	0.08	0.11	0.03	0.14	-0.09
Birth order (1st order)	2nd and higher	-0.23	**-0.20**<br>*	-0.11	0.01	0.04	0.03	-0.17	-0.03	-0.09	-0.00	0.10	-0.11	-0.09	0.15
Current weight-for-length z-score		0.05	-0.01	0.03	0.01	0.04	-0.01	0.04	0.02	0.00	**0.09**<br>*	**0.07**<br>*	-0.00	**0.11**<br>**	-0.00
Number of teeth		**0.20**<br>***	0.04	0.04	**0.04**<br>**	0.02	**0.05**<br>**	**0.05**<br>*	**0.05**<br>*	**0.03**<br>**	0.03	0.02	0.00	0.03	0.01
**Motor and feeding skills (modality of reference)**															
Pacifier using (Often)	Rarely/Sometimes	0.10	0.12	-0.05	-0.05	0.06	0.07	**0.29**<br>**	0.3	0.05	-0.12	-0.02	0.17	-0.12	-0.18
	Never	0.21	-0.01	-0.02	-0.05	-0.13	0.11	0.04	0.09	-0.09	0.06	0.12	0.00	0.11	-0.14
Thumb sucking (Sometimes/Often)	Rarely/never	0.18	0.05	0.05	0.09	0.03	0.12	0.13	0.05	0.05	-0.06	-0.06	0.01	-0.16	0.28
Eating with finger (Sometimes/Often)	Rarely/never	**-0.72**<br>**	-0.17	0.13	**-0.26**<br>***	**-0.30**<br>***	**-0.36**<br>***	**-0.47**<br>***	**-0.40**<br>***	**-0.49**<br>***	**-0.71**<br>***	**-0.57**<br>***	-0.14	-0.10	0.00
Gagging (Never)	Sometimes/Often	-0.24	**-0.20**<br>**	-0.04	-0.03	-0.05	-0.16	-0.22	-0.10	**-0.51**<br>***	**-0.64**<br>***	**-0.28**<br>*	**-0.47**<br>**	**-0.75**<br>***	**-0.37**<br>*
	Rarely	-0.26	**-0.19**<br>*	-0.01	-0.03	-0.08	-0.02	**-0.26**<br>*	-0.03	**-0.16**<br>*	**-0.22**<br>*	**-0.25**<br>**	-0.04	**-0.21**<br>*	**-0.33**<br>*
Self-feeding with a fork (Sometimes/Often)	Rarely/never	§	§	§	§	§	§	§	§	**-0.66**<br>***	**-0.49**<br>***	**-0.38**<br>***	**-0.39**<br>***	§	§
Sitting alone (Sometimes/Often)	Rarely/never	**-0.43**<br>***	**-0.25**<br>***	**-0.12**<br>*	-0.04	-0.02	§	§	§	§	§	§	§	§	§

Drooling (Sometimes/Often)	Rarely/never	§	§	0.09	0.07	-0.03	0.01	0.08	0.07	-0.01	0.13	-0.04	**0.25** *	0.14	-0.08
**Feeding practices (modality of reference)**															
Breastfeeding (Yes)	No	0.07	0.07	0.04	-0.06	-0.07	**-0.03** **	-0.28	**-0.07** *	-0.16	-0.12	0.03	**-0.13** *	-0.20	-0.11
Age of CF		-0.05	**-0.26** ***	-0.07	**-0.09** **	-0.08	-0.04	**-0.13** *	**-0.13** **	-0.02	**-0.07** *	0.01	0.09	-0.02	0.01
T1 texture exposure		**0.05** ***	**0.03** ***	**0.01** *	**0.01** **	0.01	0.00	0.00	**0.01** **	0.02	-0.00	-0.00	-0.01	-0.01	-0.02
T2 texture exposure		**0.12** ***	**0.06** ***	**0.08** ***	**0.07** ***	**0.07** ***	**0.06** ***	**0.07** ***	**0.04** ***	**0.06** ***	**0.05** ***	**0.03** ***	**0.03** **	**0.03** ***	**0.02** *
T3 texture exposure		**0.07** ***	**0.04** ***	**0.05** ***	**0.05** ***	**0.04** ***	**0.04** ***	**0.04** ***	**0.03** ***	**0.03** ***	**0.02** ***	**0.02** ***	**0.01** ***	**0.02** ***	**0.01** ***
Attendance to daycare meal		0.04	0.08	-0.03	-0.10	-0.03	0.07	0.00	-0.11	0.10	0.00	-0.07	0.08	-0.02	-0.07
Meal taken with the family (No)	Yes	**0.44****	0.14	0.08	**0.22*****	**0.27*****	**0.25***	**0.34*****	0.05	**0.22****	**0.37*****	**0.47*****	**0.33****	**0.65*****	0.29
Food Preparation types (both ready-prepared baby foods and homemade foods)	Exclusive use of ready-prepared baby food	-0.02	-0.03	-0.04	-0.07	-0.15	-0.06	**-0.23** **	-0.13	-0.27 **	-0.41 *	-0.33	§	§	§
	Exclusive use of homemade food and/or non-specific foods	-0.01	0.10	0.12	**0.17** *	**0.32** **	0.01	**0.24** **	0.18	**0.43** ***	**0.47** ***	**0.28** ***	**0.61** ***	**0.56** ***	0.23
Mother feeling / introduction of solids (unconcerned)	Eager	0.08	0.10	0.02	-0.02	-0.03	0.06	**-0.39** ***	0.04	**-0.29** **	-0.01	-0.20	0.00	-0.01	-0.03
	Reluctant	0.26	-0.05	-0.11	-0.11	-0.08	-0.16	**-0.54** ***	0.12	**-0.45** ***	**-0.35** ***	**-0.40** ***	**-0.46** ***	-0.21	**-0.32** *

§': not tested because sample size was insufficient : the percentages of children self-feeding with a fork before 13 months. drooling at 4/5 and 6. sitting up alone after 9 months and fed exclusively with ready-prepared baby foods' after 22 months were all < to 5% of the age class effective (see Table 1).
